# Supplementary material for: Extrapulmonary tuberculosis in HIV-infected patients in rural Tanzania: The prospective Kilombero and Ulanga antiretroviral cohort
Source: PLoS One. 2020 Mar 4;15(3):e0229875. doi: 10.1371/journal.pone.0229875 (PMC7055864; doi:10.1371/journal.pone.0229875)
Supplement: S1 Table — (DOCX) [file pone.0229875.s002.docx]

**Table S1: Definitions of tuberculosis**

| **Microbiologically Confirmed Tuberculosis** | |
| --- | --- |
| **Microbiologically confirmed PTB** | Presence of acid-fast bacilli or positive Xpert® MTB/RIF from a sputum  OR  A15.0-9 (TB of the lung, intrathoracic lymph nodes, trachea or bronchus confirmed bacteriologically or histologically) except A15.6 and start of TB treatment 1 week before until 3 months after working diagnosis |
| **Microbiologically confirmed EPTB** | Presence of acid-fast bacilli or positive Xpert® MTB/RIF from a sterile site (not sputum)  OR  A15.6 (Tuberculous pleurisy, confirmed bacteriologically and histologically) and start of TB treatment 1 week before until 3 months after working diagnosis |
|  |  |
| **Combined microbiologically confirmed PTB and EPTB** | Presence of acid-fast bacilli/positive Xpert® MTB/RIF from sputum and A15.6, A17 (TB of nervous system), A18 (TB of other organs), A19 (miliary TB)  OR  Presence of acid-fast bacilli/positive Xpert® MTB/RIF from sterile site and cough or infiltrate on chest x-ray |
| **Clinically diagnosed Tuberculosis** | |
| **Clinical PTB** | A16.0-9 (Tuberculosis of the lung, not confirmed bacteriologically or histologically), except A16.5 (TB pleurisy without bacteriological or histological evidence) without microbiological proof  AND  start of TB treatment 1 week before until 3 months after working diagnosis |
| **Clinical EPTB** | ICD-10 codes A16.5, A17, A18, A19 without microbiological proof, without cough or infiltrate on chest x-ray  AND  Start of anti-TB-treatment 1 week before until 3 months after working diagnosis |
| **Combined clinical PTB and EPTB** | A16.5, A17, A18, A19 and cough or infiltrate on chest x-ray without microbiological proof |
