# Supplementary material for: Extrapulmonary tuberculosis in HIV-infected patients in rural Tanzania: The prospective Kilombero and Ulanga antiretroviral cohort
Source: PLoS One. 2020 Mar 4;15(3):e0229875. doi: 10.1371/journal.pone.0229875 (PMC7055864; doi:10.1371/journal.pone.0229875)
Supplement: Attachment — Submitted filename: EPTB Tanzania PONE-D-19-26934_reviewer.pdf [file pone.0229875.s003.pdf]

PLOS ONE

Extrapulmonary tuberculosis in HIV-infected patients in rural Tanzania. The prospective Kilombero and Ulanga antiretroviral cohort.

--Manuscript Draft--

**Manuscript Number:**	PONE-D-19-26934
**Article Type:**	Research Article
**Full Title:**	Extrapulmonary tuberculosis in HIV-infected patients in rural Tanzania. The prospective Kilombero and Ulanga antiretroviral cohort.
**Short Title:**	Extrapulmonary tuberculosis in HIV-infected patients in rural Tanzania.
**Corresponding Author:**	Maja Weisser<br>University Hospital of Basel<br>Basel, SWITZERLAND
**Keywords:**	extrapulmonary tuberculosis; Tuberculosis; HIV; Xpert MTB/RIF; sub-Sahara Africa; ART; antiretroviral treatment; KIULARCO; Tanzania; rural
**Abstract:**	Background<br>In sub-Saharan Africa, diagnosis and management of extrapulmonary tuberculosis (EPTB) in people living with HIV (PLHIV) remains a major challenge. This study aimed to characterize the epidemiology and clinical outcomes of extrapulmonary tuberculosis in people living with HIV (PLHIV) in a rural setting in Tanzania.<br>Methods<br>We included PLHIV >18 years of age enrolled into the Kilombero and Ulanga antiretroviral cohort (KIULARCO) from 2013 to 2017. We assessed the diagnosis of tuberculosis by integrating prospectively collected clinical and microbiological data. We calculated prevalence- and incidence rates and used Cox regression analysis to evaluate the association of risk factors in extrapulmonary tuberculosis (EPTB) with a combined endpoint of lost to follow-up (LTFU) and death.<br>Results<br>We included 3,129 subjects (64.5% female) with a median age of 38 years (interquartile range [IQR] 31-46) and a median CD4+ cell count of 229/µl (IQR 94-421) at baseline. During the median follow-up of 1.25 years (IQR 0.46-2.85), 574 (18.4%) subjects were diagnosed with tuberculosis, whereof 175 (30.5%) had an extrapulmonary manifestation. Microbiological evidence by AFB stain or Xpert® MTB/RIF was present in 178/483 (36.9%) patients with pulmonary and in 28/175 (16.0%) of patients with extrapulmonary manifestations, respectively. Incidence density rates for pulmonary Tuberculosis (PTBI and EPTB were 17.9/1000py (95% CI 14.2-22.6) and 5.8/1000py (95% CI 4.0-8.5), respectively. The combined endpoint of death and LTFU was observed in 1058 (33.8%) patients, most frequently in the subgroup of EPTB (47.2%). The adjusted hazard ratios for death/LTFU in EPTB patients were significantly increased for patients aged >45 years (HR 1.95, 95%CI 1.15-3.3; p = 0.013), whereas ART use was protective (HR 0.15, 95% CI 0.08-0.27, p <0.001).<br>Conclusions<br>Extrapulmonary tuberculosis was a frequent manifestation in this cohort of PLHIV. The diagnosis of EPTB in the absence of histopathology and mycobacterial culture remains challenging even with availability of Xpert® MTB/RIF. Patients with EPTB had increased rates of mortality and LTFU despite early recognition of the disease after enrollment.
**Order of Authors:**	Armon Arpagaus
	Fabian Franzeck
	George Sikalengo
	Robert Christopher Ndege
	Farida Bani
	Omary Ngome Rajab
	Dorcas Mnzava

Powered by Editorial Manager® and ProduXion Manager® from Aries Systems Corporation

	Martin Rohacek
	Jerry Hella
	Klaus Reither
	Manuel Battegay
	Tracy Renee Glass
	Daniel Paris
	Maja Weisser
**Additional Information:**	
**Question**	**Response**
**Financial Disclosure**<br><br>Enter a financial disclosure statement that describes the sources of funding for the work included in this submission. Review the submission guidelines for detailed requirements. View published research articles from *PLOS ONE* for specific examples.<br><br>This statement is required for submission and **will appear in the published article** if the submission is accepted. Please make sure it is accurate.<br><br>**Unfunded studies**<br>Enter: *The author(s) received no specific funding for this work.*<br><br>**Funded studies**<br>Enter a statement with the following details:<br>• Initials of the authors who received each award<br>• Grant numbers awarded to each author<br>• The full name of each funder<br>• URL of each funder website<br>• Did the sponsors or funders play any role in the study design, data collection and analysis, decision to publish, or preparation of the manuscript?<br>• **NO** - Include this sentence at the end of your statement: *The funders had no role in study design, data collection and analysis, decision to publish, or preparation of the manuscript.*<br>• **YES** - Specify the role(s) played.<br><br>* typeset	This research was supported by grants from the Freie Akademische Gesellschaft Basel (https://fag-basel.ch).<br><br>The funders had no role in study design, data collection and analysis, decision to publish, or preparation of the manuscript.
**Competing Interests**	The authors have declared that no competing interests exist.

Use the instructions below to enter a competing interest statement for this submission. On behalf of all authors, disclose any competing interests that could be perceived to bias this work—acknowledging all financial support and any other relevant financial or non-financial competing interests.<br><br>This statement **will appear in the published article** if the submission is accepted. Please make sure it is accurate. View published research articles from *PLOS ONE* for specific examples.<br><br>**NO authors have competing interests**<br><br>Enter: *The authors have declared that no competing interests exist.*<br><br>**Authors with competing interests**<br><br>Enter competing interest details beginning with this statement:<br><br>*I have read the journal's policy and the authors of this manuscript have the following competing interests: [insert competing interests here]*<br><br>* typeset	
**Ethics Statement**<br><br>Enter an ethics statement for this submission. This statement is required if the study involved:<br><br>• Human participants<br>• Human specimens or tissue<br>• Vertebrate animals or cephalopods<br>• Vertebrate embryos or tissues<br>• Field research<br><br>Write "N/A" if the submission does not require an ethics statement.<br><br>General guidance is provided below. Consult the submission guidelines for detailed instructions. **Make sure that all**	All participants involved in this study gave written informed consent for the collection, storage and use of clinical data for research purposes within KIULARCO (Kilombero and Ulanga antiretroviral cohort). The study protocol of KIULARCO was approved by the Ifakara Health Institute Institutional Review Board, the National Institute of Medical Research in Tanzania, the Tanzanian Commission of Science and Technology as well as the Ethics committee of Northwest and Central Switzerland.

Powered by Editorial Manager® and ProduXion Manager® from Aries Systems Corporation

information entered here is included in the Methods section of the manuscript.<br><br>**Format for specific study types**<br><br>**Human Subject Research (involving human participants and/or tissue)**<br>• Give the name of the institutional review board or ethics committee that approved the study<br>• Include the approval number and/or a statement indicating approval of this research<br>• Indicate the form of consent obtained (written/oral) or the reason that consent was not obtained (e.g. the data were analyzed anonymously)<br><br>**Animal Research (involving vertebrate animals, embryos or tissues)**<br>• Provide the name of the Institutional Animal Care and Use Committee (IACUC) or other relevant ethics board that reviewed the study protocol, and indicate whether they approved this research or granted a formal waiver of ethical approval<br>• Include an approval number if one was obtained<br>• If the study involved *non-human primates*, add *additional details* about animal welfare and steps taken to ameliorate suffering<br>• If anesthesia, euthanasia, or any kind of animal sacrifice is part of the study, include briefly which substances and/or methods were applied<br><br>**Field Research**<br><br>Include the following details if this study involves the collection of plant, animal, or other materials from a natural setting:<br>• Field permit number<br>• Name of the institution or relevant body that granted permission	
**Data Availability**<br><br>Authors are required to make all data underlying the findings described fully available, without restriction, and from the time of publication. PLOS allows rare exceptions to address legal and ethical	Yes - all data are fully available without restriction

concerns. See the PLOS Data Policy and FAQ for detailed information.<br><br>A Data Availability Statement describing where the data can be found is required at submission. Your answers to this question constitute the Data Availability Statement and **will be published in the article**, if accepted.<br><br>**Important:** Stating 'data available on request from the author' is not sufficient. If your data are only available upon request, select 'No' for the first question and explain your exceptional situation in the text box.<br><br>Do the authors confirm that all data underlying the findings described in their manuscript are fully available without restriction?	
**Describe where the data may be found in full sentences. If you are copying our sample text, replace any instances of XXX with the appropriate details.**<br><br>• If the data are **held or will be held in a public repository**, include URLs, accession numbers or DOIs. If this information will only be available after acceptance, indicate this by ticking the box below. For example: *All XXX files are available from the XXX database (accession number(s) XXX, XXX.*).<br>• If the data are all contained **within the manuscript and/or Supporting Information files**, enter the following: *All relevant data are within the manuscript and its Supporting Information files.*<br>• If neither of these applies but you are able to provide **details of access elsewhere**, with or without limitations, please do so. For example:<br><br>*Data cannot be shared publicly because of [XXX]. Data are available from the XXX Institutional Data Access / Ethics Committee (contact via XXX) for researchers who meet the criteria for access to confidential data.*	All relevant data are within the manuscript and its Supporting Information files.

The data underlying the results presented in the study are available from (include the name of the third party and contact information or URL).<br>• This text is appropriate if the data are owned by a third party and authors do not have permission to share the data.<br><br>* typeset	
Additional data availability information:	

Manuscript

Click here to download Manuscript Manuscript EPBT in KIULARCO-final.docx

Extrapulmonary tuberculosis in HIV-infected patients in rural Tanzania

The prospective Kilombero and Ulanga antiretroviral cohort

Armon Arpagaus[1,2¶], Fabian Christoph Franzeck[4¶], George Sikalengo[3,5], Robert Christopher Ndege[3,5], Farida Bani[3,5], Dorcas Mnzava[3], Martin Rohacek[1,2,3], Omary Ngome Rajab[3], Jerry Hella[1,2,3], Klaus Reither[1,2], Manuel Battegay[4], Tracy Renee Glass[1,2], Daniel Henry Paris[1,2], Maja Weisser[1,3,4]* on behalf of the KIULARCO Study Group

[1] Swiss Tropical and Public Health Institute, University of Basel, Basel, Switzerland

[2] University of Basel, Basel, Switzerland

[3] Ifakara Health Institute, Ifakara, United Republic of Tanzania

[4] Division of Infectious Diseases & Hospital Epidemiology, University Hospital Basel, University of Basel, Basel, Switzerland

[5] Saint Francis Referral Hospital, Ifakara, United Republic of Tanzania

[¶]These authors have contributed equally to the work.

* *Corresponding author*

Email: m.weisser@unibas.ch (MW)

Abstract

Background

In sub-Saharan Africa, diagnosis and management of extrapulmonary tuberculosis (EPTB) in people living with HIV (PLHIV) remains a major challenge. This study aimed to characterize the epidemiology and clinical outcomes of extrapulmonary tuberculosis in people living with HIV (PLHIV) in a rural setting in Tanzania.

Methods

We included PLHIV >18 years of age enrolled into the Kilombero and Ulanga antiretroviral cohort (KIULARCO) from 2013 to 2017. We assessed the diagnosis of tuberculosis by integrating prospectively collected clinical and microbiological data. We calculated prevalence- and incidence rates and used Cox regression analysis to evaluate the association of risk factors in extrapulmonary tuberculosis (EPTB) with a combined endpoint of lost to follow-up (LTFU) and death.

Results

We included 3,129 subjects (64.5% female) with a median age of 38 years (interquartile range [IQR] 31-46) and a median CD4+ cell count of 229/µl (IQR 94-421) at baseline. During the median follow-up of 1.25 years (IQR 0.46-2.85), 574 (18.4%) subjects were diagnosed with tuberculosis, whereof 175 (30.5%) had an extrapulmonary manifestation. Microbiological evidence by AFB stain or Xpert® MTB/RIF was present in 178/483 (36.9%) patients with pulmonary and in 28/175 (16.0%) of patients with extrapulmonary manifestations, respectively. Incidence density rates for pulmonary Tuberculosis (PTB and EPTB were 17.9/1000py (95% CI 14.2-22.6) and 5.8/1000py (95% CI 4.0-8.5), respectively. The combined endpoint of death and LTFU was observed in 1058 (33.8%) patients, most frequently in the subgroup of EPTB (47.2%). The adjusted hazard ratios for death/LTFU in EPTB patients were significantly increased for patients aged >45 years (HR 1.95, 95%CI 1.15-3.3; p = 0.013), whereas ART use was protective (HR 0.15, 95% CI 0.08-0.27, p <0.001).

Conclusions

Extrapulmonary tuberculosis was a frequent manifestation in this cohort of PLHIV. The diagnosis of EPTB in the absence of histopathology and mycobacterial culture remains challenging even with availability of Xpert® MTB/RIF. Patients with EPTB had increased rates of mortality and LTFU despite early recognition of the disease after enrolment.

Introduction

Tuberculosis (TB) remains a leading cause of death, especially in patients co-infected with HIV. Of the globally 10 million patients diagnosed with TB in 2017, 9% were co-infected with HIV. Of these 72% live in Africa and contribute 84% of the 300’000 deaths [1]. Tanzania is one of the thirty high-endemic countries with an estimated TB incidence rate of 269/100’000 in the general population, an HIV-TB coinfection rate of 31% and a mortality rate in HIV co-infected patients of 39/100’000 [1].

While TB is primarily a pulmonary disease, extrapulmonary manifestations account for 14% of incident TB cases [1]. In countries with a high HIV/TB co-infection rate such as Tanzania, 20% of incident TB manifest as extrapulmonary TB (EPTB) [1]. TB can affect any organ - however, lymph nodes and pleura are the most frequent localizations [2, 3]. In African settings TB lymphadenitis has accounted for 61% - 78% and TB pleurisy for 10.6% of EPTB cases [4-6].

Diagnosis of EPTB remains challenging in resource-limited settings, as clinical manifestations are diverse, microbiological testing depends on invasive procedures and the gold standard of mycobacterial culture or supporting histopathology is mostly unavailable. The low bacillary load in tissues leads to poor sensitivity of microscopy for acid-fast bacilli (AFB) [7]. In a recent metanalysis on a wide range of different samples including samples from cerebrospinal fluid, lymph nodes and a variety tissues of patients with suspected EPTB, Xpert® MTB/RIF showed a high specificity of ≥ 98% in cerebrospinal fluid, pleural fluid, urine, and peritoneal fluid compared to culture, whereas the sensitivity ranged from 31% in pleural tissue to> 80% in urine and bone or joint fluid and tissue and 97% in bone or joint fluid compared to culture [8].

This low sensitivity of microbiological tests and the lack of diagnostic tools for important differential diagnoses of EPTB, e.g. malignancy or heart failure in case of pleural effusions [9], result in diagnostic uncertainty. As a consequence, many patients with compatible symptoms receive anti-tuberculous treatment without confirmatory testing [10], resulting in overtreatment of tuberculosis, and underdiagnosis of alternative conditions [11, 12].

Data on outcome and predictors of survival are scarce and most knowledge comes from retrospective studies. Poor outcomes have been reported in patients with central nervous manifestations [13], lymphopenia [14], older age and late presentation [15].

Our study aimed to characterize the epidemiology of EPTB in people living with HIV (PLHIV) in a rural setting in Tanzania, and to evaluate clinical outcomes and predictors for outcome.

Methods

Study site and patient population

The Kilombero and Ulanga Antiretroviral Cohort (KIULARCO) is an ongoing cohort of HIV-infected patients in care at the Chronic Diseases Clinic Ifakara, at the St. Francis Referral Hospital, located in the southwest of Tanzania. Details of the cohort have been described elsewhere [16, 17]. Since 2005, more than 10,000 consenting patients were enrolled into care with approximately 4,000 patients currently under active follow-up. Anti-tuberculous treatment, combination antiretroviral therapy (cART) and the associated care is provided free of charge by the government. On a routine basis, stable patients are assessed by a clinician 4 times per year. During clinical visits, information on demographics, clinical parameters, co-morbidities, prescription of cART and co-medications, adherence and laboratory monitoring were entered into a customized electronic clinical record system designed for subsequent scientific analysis (OpenMRS, http://openmrs.org). Xpert® MTB/RIF (Cepheid, USA) [18, 19] is available since December 2012 and was introduced in parallel to an integrated care model for HIV and TB within one facility with a standardized electronic questionnaire to detect TB signs and symptoms [20]. Since the implementation of this bundle all HIV-patients are systematically screened for TB with the World Health Organization (WHO) symptom screening tool [21]. In patients suspected of PTB, sputum smear and, in patients with EPTB, urine or a sample from a sterile site is examined by bacilloscopy and Xpert® MTB/RIF analysis. In addition, from July 2016 to June 2017, a prospective observational study on the value of sonography to rule out tuberculosis was performed at St. Francis Referral Hospital [22]. The study included HIV-positive and -negative patients with symptoms of TB and comprised performance of sonography, Xpert®MTB/RIF testing from sputum, urine samples and sterile fluids (thoracentesis, pericardiocentesis, ascites tap) improving standards for diagnosis of TB.

For this study, we included all patients aged ≥18 years enrolled into KIULARCO between September 1st, 2013 until December 31st, 2017 who completed a baseline evaluation and at least one clinical follow-up. Exclusion criteria were lack of informed consent, no follow-up visits or age <18years.

Definitions

To define PTB and EPTB, the International Classification of disease (ICD-10) [23] code and results from microbiological tests were used.

Microbiologically confirmed PTB was diagnosed, if a sputum sample was positive or an ICD-10 code for PTB (A15.x and A16.x except A15.6 and A16.5) was documented and TB treatment was initiated 1 week before until 3 months after the working diagnosis. Microbiologically confirmed EPTB was diagnosed, if acid-fast bacilli on microscopy or Xpert® MTB/RIF were positive from an extrapulmonary site or if A15.6 (TB pleurisy) and an anti-TB treatment was started 1 week before until 3 months after the working diagnosis. Microbiologically confirmed combined PTB and EPTB was defined as either a) presence of acid-fast bacilli/positive Xpert MTB/RIF from sputum and A15.6, A17, A18 or A19 or b) presence of acid-fast bacilli/positive Xpert MTB/RIF from a sterile site and cough or infiltrate on a chest x-ray without an alternative explanation.

Clinical PTB was defined as ICD-10 code A16 (excluding A16.5) without microbiological confirmation and start of TB treatment 1 week before until 3 months after working diagnosis. Clinical EPTB was defined as ICD-10 code A16.5, A17, A18, A19 without microbiological confirmation and without cough or infiltrate on chest x-ray, and start of TB treatment 1 week before until 3 months after working diagnosis

Combined clinical PTB and EPTB was defined as A16.5, A17, A18, A19 and presence of cough or infiltrate on chest x-ray without microbiological confirmation, and initiation of an anti-tuberculous treatment starting 1 week before until 3 months after an established working diagnosis.

No tuberculosis was defined as absence of positive microbiological results, ICD-10 code for Tuberculosis and no initiation of anti-tuberculosis therapy.

Cases of TB diagnosed during the first 90 days of observation after enrollment were judged as prevalent at enrolment to allow for delays in diagnoses in order to prevent over classification of incident cases.

Comorbidities were defined according to ICD-10 codes and measured variables (e.g. laboratory values or blood pressure). Anemia was defined as ICD-10 codes D50-D64.9 or by a hemoglobin level below

12g/l or 11g/l for men and women, respectively. Arterial hypertension was defined as ICD-10 codes I10 - I15 or measured blood pressure of >140/90mmHg upon two consecutive measurements or active antihypertensive treatment. Undernutrition was defined as ICD-10 codes E44-E46 or a body mass index (BMI) <18.5kg/m^2, obesity as E66.0-E66.9 or a BMI >30.0kg/m^2. Acute kidney failure was diagnosed if ICD-10 codes N17.0-17.9 were documented and chronic kidney failure as N18.0-18.9 or an uninterrupted estimated glomerular filtration rate (according to the CKD-EPI formula) <60ml/min/1.73m^2 for > 3 months.

Patients were considered being lost to follow-up (LTFU) if they did not present to the clinic within 60 days after their last scheduled appointment. We decided to use a composite clinical outcome of death and LTFU due to a significant rate of LTFU in our cohort and an assumed high mortality in those patients. [24].

Data Management and analysis

All included patients were assigned to the 4 following groups: PTB, EPTB, combined PTB and EPTB and no TB. Continuous variables were summarized using median and interquartile range and categorical variables with counts and frequencies. Comparisons of continuous and categorical variables were done using the Mann–Whitney U test and the chi-square test, respectively. Prevalence and incidence density rates per 1000 person-years (py) were calculated including confirmed and clinically diagnosed cases of tuberculosis. Kaplan–Meier estimates were used to plot cumulative survival probabilities. Univariate and multivariate Cox proportional hazards models were fitted for patients with EPTB (period starting from the date of first diagnosis of EPTB) to explore associations of a priori defined variables (sex, age, CD4+ cell count at baseline, WHO stage at baseline, cART and CNS manifestations of TB) with a composite outcome of lost to follow-up and death. The age variable was dichotomized at the upper quartile of the distribution of the cohort. The proportional hazard assumption was checked using Schoenfeld residuals. Missing data were excluded (complete case analysis). All statistical analyses were performed using Stata 12.1 (StataCorp, USA).

Ethical considerations

All participants involved in this study gave written informed consent for the collection, storage and use of clinical data for research purposes within KIULARCO. The study protocol of KIULARCO was

approved by the Ifakara Health Institute Institutional Review Board, the National Institute of Medical Research in Tanzania, the Tanzanian Commission of Science and Technology as well as the Ethics committee of Northwest and Central Switzerland.

Results

Study population

In total, 3,620 patients were enrolled into KIULARCO between January 1st 2013 and December 31st 2017. Of these, we excluded 363 patients <18 years of age and 128 patients without any follow-up visits, resulting in 3,129 patients included in the final analysis **(Fig 1)**. The median follow-up time was 1.25 years (IQR 0.46-2.85) resulting in a total of 5370 person-years of follow-up.

Fig 1. Flowchart of patients included in the study.
Patients screened and included in the study. After inclusion, patients were allocated to 4 groups: patients with no tuberculosis (no TB), patients with pulmonary Tuberculosis (PTB), patients with extrapulmonary tuberculosis (EPTB) and patients with pulmonary and extrapulmonary tuberculosis. LTFU, lost to follow-up.

Baseline characteristics of patients at the time of enrolment into KIULARCO according to TB group are shown in **Table 1**. The median age ranged from 36.5 years (IQR 31.3, 42.6) in the EPTB group to 39.9 years (IQR 34.0, 46.8) in the PTB group. The majority of patients (64.7%) were females, only the subgroup of patients with combined PTB and EPTB comprised more males (54.8%). The HIV infection at enrolment was more advanced in patients with a consecutive diagnosis of TB at any location: WHO stage 3/4 was attributed to 34.7% in the no TB group versus 79.7%, 75.9% and 85.7% of the PTB, EPTB and PTB/EPTB group, respectively. The highest median CD4+ cell count was observed in the no TB group with 262 cells/µl, whereas in all other groups the median count was <200 cells/µl (113, 159 and 128 cells/µl in the PTB, EPTB and PTB/EPTB group, respectively). There were no major differences in marital status, education, smoking behavior and alcohol consumption.

Regarding comorbidities within the first three months after enrolment, anemia was highly prevalent in all groups, but more so in patients with TB with the highest prevalence in the PTB/EPTB group with up to 85.7%. Undernutrition was also more prevalent in TB groups (no TB 14.1%, PTB 37.8%, EPTB 23.3%, PTB/EPTB 28.6%).

Table 1. Baseline Characteristics

		no TB	PTB	EPTB	PTB and EPTB
N (total=3129)		2555	399	91	84
Microbiologically confirmed TB		-	144 (36.1%)	8 (8.8%)	34 (40.4%)£, 20 (23.8%)$
Positive Xpert® MTB/RIF		-	84 (21.0%)	7 (7.7%)	16 (19%)£ / 12 (14.3%)$
Year of registration					
	2013	332 (13.0%)	57 (14.3%)	15 (16.5%)	8 (9.5%)
	2014	476 (18.6%)	112 (28.1%)	16 (17.6%)	17 (20.2%)
	2015	630 (24.7%)	133 (33.3%)	26 (28.6%)	32 (38.1%)
	2016	548 (21.4%)	64 (16.0%)	17 (18.7%)	9 (10.7%)
	2017	569 (22.3%)	33 (8.3%)	17 (18.7%)	18 (21.4%)
Age, years, median (IQR)		38.0 (31.2, 46.2)	39.9 (34.0, 46.8)	36.5 (31.3, 42.6)	39.9 (33.6, 45.5)
Sex, female		1716 (67.2%)	214 (53.6%)	55 (60.4%)	38 (45.2%)
Marital status					
	Married/living in relationship	1581 (61.9%)	205 (51.4%)	54 (59.3%)	54 (64.3%)
	Living alone	974 (38.1%)	194 (48.6%)	37 (40.7%)	30 (35.7%)
Smoking, current		102 (4.0%)	37 (9.3%)	8 (8.8%)	7 (8.3%)
	Missing information	233 (9.1%)	14 (3.5%)	2 (2.2%)	2 (2.4%)
Alcohol consumption, current		297 (11.6%)	67 (16.8%)	14 (15.4%)	9 (10.7%)
	Missing information	233 (9.1%)	14 (3.5%)	2 (2.2%)	2 (2.4%)
HIV WHO stage					
	1	1086 (42.5%)	45 (11.3%)	14 (15.4%)	9 (10.7%)
	2	385 (15.1%)	29 (7.3%)	7 (7.7%)	2 (2.4%)
	3	588 (23.0%)	238 (59.6%)	27 (29.7%)	37 (44.0%)
	4	299 (11.7%)	80 (20.1%)	42 (46.2%)	35 (41.7%)
	Missing information	197 (7.7%)	7 (1.8%)	1 (1.1%)	1 (1.2%)
CD4+ cell count (/µl)					
	<100	459 (18.0%)	160 (40.1%)	30 (33.0%)	29 (34.5%)
	100-199	387 (15.1%)	67 (16.8%)	21 (23.1%)	20 (23.8%)
	200-499	837 (32.8%)	83 (20.8%)	25 (27.5%)	21 (25.0%)
	>=500	439 (17.2%)	33 (8.3%)	7 (7.7%)	2 (2.4%)
	Missing information	433 (16.9%)	56 (14.0%)	8 (8.8%)	12 (14.3%)
CD4+ count (/µl), median (IQR)		262 (117, 450)	113 (49, 280)	159 (60, 303)	128 (54, 220)
Comorbidities					
	Anemia	1189 (46.6%)	292 (73.2%)	72 (80.0%)	72 (85.7%)
	Thrombocytopenia	300 (11.8%)	58 (14.5%)	15 (16.7%)	13 (15.5%)
	Undernutrition	359 (14.1%)	151 (37.8%)	21 (23.3%)	24 (28.6%)
	Obesity	136 (5.3%)	11 (2.8%)	1 (1.1%)	2 (2.4%)
	Arterial Hypertension	377 (14.8%)	40 (10.0%)	10 (11.1%)	10 (11.9%)
	Cryptococcosis	56 (2.2%)	8 (2.0%)	3 (3.3%)	1 (1.2%)
	Viral hepatitis	140 (5.5%)	26 (6.5%)	4 (4.4%)	6 (7.1%)
	Acute kidney failure	33 (1.3%)	9 (2.3%)	5 (5.6%)	6 (7.1%)
	Chronic kidney failure	21 (0.8%)	2 (0.5%)	0 (0.0%)	2 (2.4%)
	Malaria	206 (8.1%)	55 (13.8%)	8 (8.9%)	10 (11.9%)
	Lower respiratory tract infection	195 (7.6%)	60 (15.0%)	4 (4.4%)	7 (8.3%)

EPTB, extrapulmonary tuberculosis; IQR, interquartile range; PTB, pulmonary tuberculosis; TB, tuberculosis; WHO, world health organisation

£ positive sputum sample; $ positive extrapulmonary sample

Prevalence and incidence of PTB, EPTB and combined PTB/EPTB

Overall, 574 (18.4%) patients were diagnosed with confirmed or clinical TB at any localization (**Fig 1**). An extrapulmonary manifestation was observed in 175 (5.6%) patients, whereof 84 (48%) had concurrent pulmonary TB (PTB and EPTB group) and 91 (52%) no signs of pulmonary TB (EPTB group). Exclusive pulmonary manifestations were documented in 399 (12.8%) patients (PTB group).

The diagnosis of EPTB was made a median of 3.5 (IQR 1-21.5) days and PTB at 2 (IQR 1-17) days after enrolment. Most cases of TB were diagnosed during the first 90 days of observation and thus classified as prevalent at enrolment: this was the case in 334 (83.7%) in PTB, 78 (85.7%) in EPTB and 71 (84.5%) in PTB/EPTB. Tuberculosis diagnoses occurring 3 months or later after enrolment (incident cases) were diagnosed at an incidence density rate of 17.9/1000py (95% CI 14.2-22.6) for PTB and 5.8/1000py (95% CI 4.0-8.5) for EPTB.

Analyzing the time point of diagnosis of TB in relation to start of cART, in the majority of patients (406/508; 71.8%) TB was diagnosed before start of cART (**Fig 2**). No further information from the clinical notes concerning possible TB-associated immune reconstitution inflammatory syndrome (TB-IRIS) was available. In the subset of patients with initiation of cART ahead of a diagnosis of an incident case of TB, the median time from initiation of cART to the diagnosis of PTB and EPTB was 285 days (IQR 128-659) and 305 days (IQR 114-752), respectively, possibly representing unmasking IRIS cases.

Fig 2. Interval between start of antiretroviral treatment and diagnosis of tuberculosis. (A) extrapulmonary tuberculosis (EPTB), (B) pulmonary tuberculosis (PTB).

Clinical presentation and microbiological testing

Affected organs in the 175 patients (multiple per patient possible) diagnosed with EPTB or PTB/EPTB were pleural space in 42 (24.0%), lymph node in 41 (23.4%), pericardium in 32 (18.3%), central nervous system in 21 (12%), urogenital tract in 18 (10.3%), peritoneum in 17 (9.7%), miliary in 13 (7.4%), bone in 2 (1.1%) and other in 21 (12.0%) of patients.

In total, 178/483 (36.9%) of patients with a pulmonary manifestation and 28/175 (16.0%) of patients with an extrapulmonary manifestation were confirmed microbiologically **(Table 1)**.

In total, we tested 1,241 samples (of 807 patients) with Xpert® MTB/RIF, whereof 500 (40.2%) were of extrapulmonary origin. Overall, 157/1241 (12.7%) samples analyzed were positive. Sputum samples were positive in 132/741 (17.8%) samples, extrapulmonary samples in 25/500 (5.0%) samples (**Table 2**). Over the study period, the number of performed Xpert® MTB/RIF tests per year increased for all materials with maximum usage in 2017 (**Table S2**).

Table 2. Count and Proportion of positive GeneXpert MTB/RIF by patient group (multiple tests per patients possible)

	**No TB**	**PTB**	**EPTB**	**PTB and EPTB**	**Total**
Sputum	0/396	106/275 (38.5%)	0/28	26/42 (61.9%)	132/741 (17.8%)
CSF	0/72	0/12	2/10 (20%)	0/9	2/103 (1.9%)
Pleural fluid	0/0	0/9	0/6	5/18 (27.7%)	5/33 (15.2%)
Ascitic fluid	0/11	0/0	1/4 (25%)	0/3	1/18 (5.6%)
Urine	0/188	0/85	4/24 (16%)	13/46 (28.3%)	17/343 (5%)
Lymphnode	0/2	0/0	0/0	0/1	0/3

multiple tests per patient possible

CSF, cerebrospinal fluid; EPTB, extrapulmonary tuberculosis; PTB, pulmonary tuberculosis; TB, tuberculosis

Treatment and Outcome

Combined antiretroviral therapy (cART) according to national guidelines was initiated in 2,786 (89.0%) of included patients a median of 6 (IQR 1-19, 90th percentile 102) days after enrolment. Out of the 574 patients with any diagnosis of TB, 508 (88.5%) started cART after a median of 23 (IQR 10-56) days. In the subset of patients with EPTB (including PTB/EPTB), 148 (82%) started cART after a median of 20 (IQR 7-40) days after enrolment. The most frequent combinations of antiretrovirals initiated were tenofovir disoproxil/lamivudine/efavirenz in 1,911 (68.5%) and tenofovir disoproxil/emtricitabine/efavirenz in 423 (15.2%) of patients started on cART.

Table 3 and **Figure 3** show the numbers of patients who died or were lost to follow-up (LTFU). Overall, 154 (4.9%) patients died (14.3% of patients with EPTB, 8.5% of patients with PTB and 3.7% of patients without TB). The combined endpoint of death/LTFU was highest in patients with EPTB (47.2% for EPTB alone, 46.4% for PTB/EPTB).

Fig 3. Kaplan-Meier curves for composite outcome of lost to follow-up/dead in patients with tuberculosis.

Table 3. Composite outcome of death/LTFU

	**No TB** (n=2555)	**PTB** (n= 399)	**EPTB** (n=91)	**EPTB and PTB** (n=84)	**p-value**
Dead/LTFU	839 (32.8%)	137 (34.3%)	43 (47.2%)	39 (46.4%)	0.003
Dead	95 (3.7%)	34 (8.5%)	13 (14.3%)	12 (14.3%)	<0.001

EPTB, extrapulmonary tuberculosis; LTFU, lost to follow-up; PTB, pulmonary tuberculosis; TB, tuberculosis

Cox proportional hazard models were fitted using the data of all 175 with an EPTB (EPTB and PTB/EPTB group) to analyze factors associated with the composite outcome of death and LTFU (**Table 4**). In both the univariable and multivariable model, patients with EPTB, who have ever received cART had a significantly decreased risk of death/LTFU compared to patients who were not yet on cART (HR 0.15, 95% CI 0.08-0.27, p <0.001). The multivariable hazard ratios for death/LTFU were significantly increased for patients aged >45 years (HR 1.95, 95%CI 1.15-3.3; p = 0.013).

Table 4. Cox proportional hazard model for composite outcome of LTFU/death in EPTB Patients (with or without pulmonary manifestation)

		Univariable		Multivariable	
		HR (95% CI)	*p*	*HR (95% CI)*	*p*
Female sex		0.86 (0.52, 1.41)	0.541	0.86 (0.52, 1.41)	0.541
CD4+ cell count at baseline [cells/ul]					
	<200	*Reference*		*Reference*	
	≥200	0.87 (0.53, 1.42)	0.59	0.94 (0.56, 1.58)	0.829
WHO stage at baseline					
	I & II	*Reference*		*Reference*	
	III & IV	1.10 (0.62, 1.97)	0.743	0.88 (0.46, 1.67)	0.693
Tuberculous meningitis		0.91 (0.44, 1.89)	0.805	0.71 (0.33, 1.50)	0.363
ART intake		0.19 (0.11, 0.32)	<0.001	0.15 (0.08, 0.27)	<0.001
Age > 45 years		1.35 (0.84, 2.16)	0.214	1.95 (1.15, 3.30)	0.013

ART, antiretroviral therapy; CI, confidence intervall; HR, hazard ratio; LTFU, lost to follow-up; WHO world health organization

Discussion

In this rural sub-Saharan African cohort of PLHIV we found an EPTB prevalence of 5.6% with a high mortality of 14.3% (25/175) and a combined death/LTFU rate of 46.8%. Diagnosis remains a major challenge as only 36.9% of all pulmonary TB manifestations and 16% of extrapulmonary manifestations were confirmed by microbiology. Patients with EPTB not receiving cART and with an age >45 years had a higher risk for a poor outcome.

The overall TB prevalence of 18.4% in our study is comparable to the UNAIDS Report 2018 showing a TB prevalence of 16% in PLHIV [25]. However, the 30% of EPTB within TB cases is considerably higher than rates documented worldwide and in Tanzania (14%, 22%, respectively) [1]. Studies from other rural sub-Saharan African settings, e.g. Ethiopia, also found a high EPTB prevalence in PLHIV (36.8%) [26]. Reasons might be the high number of patients presenting with advanced HIV (65%) and co-factors such as undernutrition [27, 28], which has been demonstrated to be 30% in the same cohort [29] and is more prevalent in rural than in urban settings [30]. Over the study duration, the proportion of patients with a diagnosis of EPTB in all patients with TB increased from 28.8% in 2013 to 51% in 2017 patients, which might be rather due to a reporting bias and not a real increase, as we did concurrently a prospective study on the impact of sonography for EPTB diagnosis, leading to better evaluation of patients with TB [22]. In other settings implementation of cART in has led to a decrease in EPTB in PLHIV [31].

In our study, the rate of positive Xpert® MTB/RIF was low with 12.7% (157/1,241 samples analyzed). While the majority of positive samples (86.3%) originated from sputum, the rate of positive samples from extrapulmonary sites was very low with 5% (25/500). Poor sensitivity of Xpert® MTB/RIF has been demonstrated in PLHIV with HIV previously [5] and is partly due to challenges in proper sampling [32] and the paucibacillary nature of disease in PLHIV and in extrapulmonary sites [33, 34]. In one study overall sensitivity and specificity of Xpert® MTB/RIF compared to culture was 81.3% and 99.8%, respectively in patients with EPTB. However, when compared to a combination of culture and clinical diagnosis, the sensitivity of Xpert® MTB/RIF in cavitary fluids was under 50% [35]. The inclusion of the clinical diagnosis into a combined gold standard is based on post-mortem studies in PLHIV in resource-limited settings, which have shown a prevalence of TB in sub-Saharan Africa up to 43.2% [36]. A recent meta-analysis found a sensitivity of Xpert® MTB/RIF in pleural fluid of 50.9% and documents the

important role of Xpert® MTB/RIF as confirmatory test in extrapulmonary samples, while exclusion remains a challenge [8].

While the majority of TB cases (71.8%) were diagnosed before the start of cART, a minority (9.1%) were diagnosed in first 30 days after, possibly presenting an unmasking IRIS. This hyper-responsiveness due to an increase in T-cells [37] has been reported to occur in 36-54% of TB/HIV co-infected patients [38, 39] and usually occurs within the first month after start of the antiretroviral therapy [40]. Patients presenting late after cART start with incident TB were few – unfortunately we could not analyze details on cART adherence or virological suppression as routine viral load was not implemented during the time of the study.

In a meta-analysis of PLHIV in sub-Saharan Africa around 40% of patients were LTFU five years after initiation of cART with death rates approaching 15% [41]. In our cohort this number was even higher in those with EPTB diagnosis. A recent meta-analysis including data of 7,377 patients who started cART and were subsequently LTFU showed a rate of 21.8% of subjects to be deceased while 14.8% of patients LTFU have been transferred to another clinic [42]. Factors associated with poor outcome in patients with EPTB in our study were age of >45 years and not being on cART. Both confirm previous studies, where age has been shown as a risk factor for disseminated TB and death [43, 44], while patients with EPTB co-infected with HIV without cART showed poorer outcomes than with cART [4].

A major strength of this study is the prospective assessment of TB by means of ICD-10 codes in a long-term cohort of PLHIV ensuring standardized reporting. Furthermore, this study depicts a sub-Saharan African rural setting representative of settings with limited diagnostic resources available to clinicians and a high proportion of diagnoses relying on clinical presentation only. Those conditions are neglected as many studies have been done in urban settings.

The clinical diagnosis of EPTB, however, determined by the physician also remains a major limitation, as it is a poorly defined entity, might have been assessed differently by physicians. Patients with important differential diagnoses such as cancer, heart failure, lung abscess or other diseases might have been missed. As in other studies from African settings [4, 5, 45, 46] the most frequent sites of EPTB were pleurisy and lymphadenopathy. As the lymphadenopathy was a clinical diagnosis without histological confirmation, the assumption of TB as underlying disease might have been over estimated.

Besides improving microbiological detection of TB, e.g. by Xpert MTB/RIF Ultra [47], lateral flow urine lipoarabinomannan assay (LF-LAM) [48] or adenosin deaminase [49, 50], the clinical evaluation might benefit from integration of an additional standardized testing, such as e.g. Focused Assessment with Sonography for HIV and Tuberculosis (FASH) [22, 51] .
Another limitation is the rather low observation period of patients after start on cART (median 1.25years), mainly due to the high number of patients LTFU, which precluded an exact estimation of the true mortality rate.

In conclusion, EPTB remains a relevant comorbidity with a high mortality and LTFU in a rural sub-Sahara African setting. Establishing a microbiological diagnosis of EPTB in the absence of culture and histopathology remains a major challenge in settings, where invasive procedures are not routinely performed. Despite the increasing access to Xpert® MTB/RIF MTB/RIF in sub-Saharan Africa it remains unclear to what extent this translates in clinical benefit [52]. Lack of ART and age >45 years were risk factors for death or loss to follow-up. Further research needs to be conducted to better define clinical predictors and new microbiological tests to improve early detection and outcome of EPTB.

Acknowledgments

We thank all patients who participated in KIULARCO and all staff members of the Chronic Disease Clinic at the St. Francis Referral Hospital, Ifakara, Tanzania. We thank the Government of the Canton of Basel, Switzerland, the Swiss Tropical & Public Health Institute, the Ifakara Health Institute, the University Hospital Basel, the Government of Tanzania, and the United States Agency for International Development through TUNAJALI-Deloitte/USAID Boresha Afya for support of the CDCI.

Authors' contributions

AA and MW conceived of the study. All authors participated in the design and the data collection of the study. FCF carried out the analysis of cohort data. AA, FCF and MW drafted the article. GS, RN, DM, MR, JH, KR, MB, TRG and DHP contributed to writing the manuscript. All authors edited and approved the final article.

References

1. WHO, Anderson L, Baddeley A, Dias HM, Floyd K, Baena IG. Global Tuberculosis Report 2018. 2018.
2. Sharma SK, Mohan A. Extrapulmonary tuberculosis. The Indian journal of medical research. 2004;120(4):316-53. Epub 2004/11/03. PubMed PMID: 15520485.
3. Tatar D, Senol G, Alptekin S, Gunes E, Aydin M, Gunes O. Assessment of Extrapulmonary Tuberculosis in Two Provinces of Turkey. Iran J Public Health. 2016;Vol. 45(No.3, Mar 2016):pp.305-13.
4. Ade S, Harries AD, Trebucq A, Ade G, Agodokpessi G, Adjonou C, et al. National profile and treatment outcomes of patients with extrapulmonary tuberculosis in Benin. PLoS One. 2014;9(4):e95603. Epub 2014/04/24. doi: 10.1371/journal.pone.0095603. PubMed PMID: 24755603; PubMed Central PMCID: PMCPMC3995824.
5. Fanosie A, Gelaw B, Tessema B, Tesfay W, Admasu A, Yitayew G. Mycobacterium tuberculosis Complex and HIV Co-Infection among Extrapulmonary Tuberculosis Suspected Cases at the University of Gondar Hospital, Northwestern Ethiopia. PLoS One. 2016;11(3):e0150646. Epub 2016/03/08. doi: 10.1371/journal.pone.0150646. PubMed PMID: 26950547; PubMed Central PMCID: PMCPMC4780813.
6. Alemie GA, Gebreselassie F. Common types of tuberculosis and co-infection with HIV at private health institutions in Ethiopia: a cross sectional study. BMC Public Health. 2014;14:319. Epub 2014/04/09. doi: 10.1186/1471-2458-14-319. PubMed PMID: 24708793; PubMed Central PMCID: PMCPMC4234020.
7. Angela Houston DCM. Extrapulmonary tuberculosis. Elsevier Medicine. 2014;42:1. doi: 10.1016/j.mpmed.2013.10.008.
8. Kohli M, Schiller I, Dendukuri N, Dheda K, Denkinger CM. Xpert((R)) MTB/RIF assay for extrapulmonary tuberculosis and rifampicin resistance. The Cochrane database of systematic reviews. 2018;8:Cd012768. Epub 2018/08/28. doi: 10.1002/14651858.CD012768.pub2. PubMed PMID: 30148542.
9. Porcel JM, Aleman C, Bielsa S, Sarrapio J, Fernandez de Sevilla T, Esquerda A. A decision tree for differentiating tuberculous from malignant pleural effusions. Respir Med. 2008;102(8):1159-64. Epub 2008/06/25. doi: 10.1016/j.rmed.2008.03.001. PubMed PMID: 18573650.
10. WHO, Apers L, Blanc L, Bloom A, Chakaya J, Corbett L, et al. Improving the diagnosis and treatment of smear-negative pulmonary and extrapulmonary tuberculosis among adults and adolescents - Recommendations for HIV-prevalent and resource-constrained settings. 2007.
11. Richter C, Ndosi B, Mwammy AS, Mbwambo RK. Extrapulmonary tuberculosis--a simple diagnosis? A retrospective study at Dar es Salaam, Tanzania. Tropical and geographical medicine. 1991;43(4):375-8. Epub 1991/10/01. PubMed PMID: 1812603.
12. Theron G, Peter J, Dowdy D, Langley I, Squire SB, Dheda K. Do high rates of empirical treatment undermine the potential effect of new diagnostic tests for tuberculosis in high-burden settings? The Lancet Infectious diseases. 2014;14(6):527-32. Epub 2014/01/21. doi: 10.1016/s1473-3099(13)70360-8. PubMed PMID: 24438820.
13. Kim JH, Langston AA, Gallis HA. Miliary tuberculosis: epidemiology, clinical manifestations, diagnosis, and outcome. Reviews of infectious diseases. 1990;12(4):583-90. Epub 1990/07/01. doi: 10.1093/clinids/12.4.583. PubMed PMID: 2385765.
14. Maartens G, Willcox PA, Benatar SR. Miliary tuberculosis: rapid diagnosis, hematologic abnormalities, and outcome in 109 treated adults. The American journal of medicine. 1990;89(3):291-6. Epub 1990/09/01. doi: 10.1016/0002-9343(90)90340-j. PubMed PMID: 2393033.
15. Sahn SA, Neff TA. Miliary tuberculosis. The American journal of medicine. 1974;56(4):495-505. doi: 10.1016/0002-9343(74)90482-3.
16. Letang E, Kalinjuma AV, Glass TR, Gamell A, Mapesi H, Sikalengo GR. Cohort profile: The Kilombero and Ulanga Antiretroviral Cohort (KIULARCO) - A prospective HIV cohort in rural Tanzania. Swiss medical weekly. 2017;147:w14485. Epub 2017/07/12. doi: 10.4414/smw.2017.14485. PubMed PMID: 28695551.
17. Vanobberghen F, Letang E, Gamell A, Mnzava DK, Faini D, Luwanda LB, et al. A decade of HIV care in rural Tanzania: Trends in clinical outcomes and impact of clinic optimisation in an open, prospective cohort. PLoS One. 2017;12(7):e0180983. Epub 2017/07/19. doi: 10.1371/journal.pone.0180983. PubMed PMID: 28719610; PubMed Central PMCID: PMCPMC5515476.
18. Blakemore R, Story E, Helb D, Kop J, Banada P, Owens MR, et al. Evaluation of the analytical performance of the Xpert MTB/RIF assay. J Clin Microbiol. 2010;48(7):2495-501. Epub 2010/05/28. doi: 10.1128/jcm.00128-10. PubMed PMID: 20504986; PubMed Central PMCID: PMCPMC2897495.
19. Boehme CC, Nabeta P, Hillemann D, Nicol MP, Shenai S, Krapp F, et al. Rapid molecular detection of tuberculosis and rifampin resistance. The New England journal of medicine. 2010;363(11):1005-15. Epub

2010/09/10. doi: 10.1056/NEJMoa0907847. PubMed PMID: 20825313; PubMed Central PMCID: PMCPMC2947799.

20. Haraka F, Glass TR, Sikalengo G, Gamell A, Ntamatungiro A, Hatz C, et al. A Bundle of Services Increased Ascertainment of Tuberculosis among HIV-Infected Individuals Enrolled in a HIV Cohort in Rural Sub-Saharan Africa. PLoS One. 2015;10(4):e0123275. Epub 2015/04/22. doi: 10.1371/journal.pone.0123275. PubMed PMID: 25897491; PubMed Central PMCID: PMCPMC4405488.
21. WHO, Ahmedov S, Ayles H, Blok L, Churchyard G, Corbett L, et al. Systematic screening for active tuberculosis. Principles and recommendations. 2013.
22. Ndege R, Weisser M, Elzi L, Diggelmann F, Bani F, Gingo W, et al. Sonography to Rule Out Tuberculosis in Sub-Saharan Africa: A Prospective Observational Study. Open forum infectious diseases. 2019;6(4):ofz154. Epub 2019/05/02. doi: 10.1093/ofid/ofz154. PubMed PMID: 31041350; PubMed Central PMCID: PMCPMC6483805.
23. WHO. International Statistical Classification of Diseases and Related Health Problems 10th Revision. 2016.
24. Vanobberghen F, Weisser M, Mkumbo J, Kasuga B, Katende A, Battegay M. Mortality rates in a cohort of people living with HIV in rural Tanzania: A novel extension to double sampling following tracing. . 16th European AIDS Conference, Milan. 2017.
25. UNAIDS. UNAIDS Data 2018. 2018.
26. Mohammed H, Assefa N, Mengistie B. Prevalence of extrapulmonary tuberculosis among people living with HIV/AIDS in sub-Saharan Africa: a systemic review and meta-analysis. HIV/AIDS (Auckland, NZ). 2018;10:225-37. Epub 2018/11/23. doi: 10.2147/hiv.S176587. PubMed PMID: 30464643; PubMed Central PMCID: PMCPMC6225852.
27. Vanobberghen F, Letang E, Gamell A, Mnzava DK, Faini D, Luwanda LB, et al. A decade of HIV care in rural Tanzania: Trends in clinical outcomes and impact of clinic optimisation in an open, prospective cohort. PLoS One. 2017;12(7):e0180983. Epub 2017/07/19. doi: 10.1371/journal.pone.0180983. PubMed PMID: 28719610; PubMed Central PMCID: PMCPMC5515476.
28. Essen Lv, Kalinjuma A, Katende A, Mapesi H, Matskiv N, Glass T, et al. Characterization of advanced HIV disease in a rural sub-Saharan African cohort. 2018;IAS Amsterdam.
29. Albrecht S, Franzeck FC, Mapesi H, Hatz C, Kalinjuma AV. Age-related comorbidities and mortality in people living with HIV in rural Tanzania. Aids. 2019;33(6):1031-41. Epub 2019/04/05. doi: 10.1097/qad.0000000000002171. PubMed PMID: 30946157.
30. Sikalengo G, Hella J, Mhimbira F, Rutaihwa LK, Bani F, Ndege R, et al. Distinct clinical characteristics and helminth co-infections in adult tuberculosis patients from urban compared to rural Tanzania. Infectious diseases of poverty. 2018;7(1):24. Epub 2018/03/28. doi: 10.1186/s40249-018-0404-9. PubMed PMID: 29580279; PubMed Central PMCID: PMCPMC5868052.
31. Hoogendoorn JC, Ranoto L, Muditambi N, Railton J, Maswanganyi M. Reduction in extrapulmonary tuberculosis in context of antiretroviral therapy scale-up in rural South Africa. Epidemiology and infection. 2017;145(12):2500-9. Epub 2017/07/28. doi: 10.1017/s095026881700156x. PubMed PMID: 28748775.
32. Mhalu G, Hella J, Doulla B, Mhimbira F, Mtutu H. Do Instructional Videos on Sputum Submission Result in Increased Tuberculosis Case Detection? A Randomized Controlled Trial. PLoS One. 2015;10(9):e0138413. Epub 2015/09/30. doi: 10.1371/journal.pone.0138413. PubMed PMID: 26418678; PubMed Central PMCID: PMCPMC4587748.
33. Theron G, Peter J, Calligaro G, Meldau R, Hanrahan C. Determinants of PCR performance (Xpert MTB/RIF), including bacterial load and inhibition, for TB diagnosis using specimens from different body compartments. Scientific Reports. 2014;4:5658. doi: 10.1038/srep05658.
34. Zeka AN, Tasbakan S, Cavusoglu C. Evaluation of the GeneXpert MTB/RIF Assay for Rapid Diagnosis of Tuberculosis and Detection of Rifampin Resistance in Pulmonary and Extrapulmonary Specimens. Journal of Clinical Microbiology. 2011;49(12):4138-41. doi: 10.1128/jcm.05434-11.
35. Tortoli E, Russo C, Piersimoni C, Mazzola E, Dal Monte P, Pascarella M. Clinical validation of Xpert MTB/RIF for the diagnosis of extrapulmonary tuberculosis. The European respiratory journal. 2012;40(2):442-7. Epub 2012/01/14. doi: 10.1183/09031936.00176311. PubMed PMID: 22241741.
36. Gupta RK, Lucas SB, Fielding KL, Lawn SD. Prevalence of tuberculosis in post-mortem studies of HIV-infected adults and children in resource-limited settings: a systematic review and meta-analysis. AIDS. 2015;29(15):1987-2002. Epub 2015/08/13. doi: 10.1097/QAD.0000000000000802. PubMed PMID: 26266773; PubMed Central PMCID: PMCPMC4568896.
37. Barber DLA, B. B.; Sereti, I.; Sher, A. Immune reconstitution inflammatory syndrome: the trouble with immunity when you had none. Nature reviews Microbiology. 2012;10(2):150-6. Epub 2012/01/11. doi: 10.1038/nrmicro2712. PubMed PMID: 22230950; PubMed Central PMCID: PMCPMC3507517.

38. Narita M, Ashkin D, Hollender ES, Pitchenik AE. Paradoxical worsening of tuberculosis following antiretroviral therapy in patients with AIDS. American journal of respiratory and critical care medicine. 1998;158(1):157-61. Epub 1998/07/09. doi: 10.1164/ajrccm.158.1.9712001. PubMed PMID: 9655723.
39. Narendran G, Andrade BB, Porter BO, Chandrasekhar C, Venkatesan P. Paradoxical tuberculosis immune reconstitution inflammatory syndrome (TB-IRIS) in HIV patients with culture confirmed pulmonary tuberculosis in India and the potential role of IL-6 in prediction. PLoS One. 2013;8(5):e63541. Epub 2013/05/22. doi: 10.1371/journal.pone.0063541. PubMed PMID: 23691062; PubMed Central PMCID: PMCPMC3656926.
40. Tweya H, Feldacker C, Mpunga J, Kanyerere H, Heller T, Ganesh P, et al. The shift in tuberculosis timing among people living with HIV in the course of antiretroviral therapy scale-up in Malawi. Journal of the International AIDS Society. 2019;22(4):e25240. doi: 10.1002/jia2.25240.
41. Haas AD, Zaniewski E, Anderegg N, Ford N, Fox MP. Retention and mortality on antiretroviral therapy in sub-Saharan Africa: collaborative analyses of HIV treatment programmes. Journal of the International AIDS Society. 2018;21(2). Epub 2018/02/27. doi: 10.1002/jia2.25084. PubMed PMID: 29479867; PubMed Central PMCID: PMCPMC5897849.
42. Chammartin F, Zurcher K, Keiser O, Weigel R, Chu K, Kiragga AN, et al. Outcomes of Patients Lost to Follow-up in African Antiretroviral Therapy Programs: Individual Patient Data Meta-analysis. Clinical infectious diseases : an official publication of the Infectious Diseases Society of America. 2018;67(11):1643-52. Epub 2018/06/12. doi: 10.1093/cid/ciy347. PubMed PMID: 29889240; PubMed Central PMCID: PMCPMC6233676.
43. Govender K, Suleman F, Moodley Y. Clinical risk factors for in-hospital mortality in older adults with HIV infection: findings from a South African hospital administrative dataset. The Pan African medical journal. 2017;26:126. Epub 2017/05/24. doi: 10.11604/pamj.2017.26.126.11000. PubMed PMID: 28533849; PubMed Central PMCID: PMCPMC5429410.
44. Qian X, Nguyen DT, Lyu J, Albers AE, Bi X, Graviss EA. Risk factors for extrapulmonary dissemination of tuberculosis and associated mortality during treatment for extrapulmonary tuberculosis. Emerging microbes & infections. 2018;7(1):102. Epub 2018/06/07. doi: 10.1038/s41426-018-0106-1. PubMed PMID: 29872046; PubMed Central PMCID: PMCPMC5988830.
45. Alemie GA GF. Common types of tuberculosis and co-infection with HIV at private health institutions in Ethiopia: a cross sectional study. 2014.
46. Shanmuganathan A, Srinivasan R, Thilagavathy G, Satishkumar D, Sidduraj C, James B. Determination of Sites Involved, HIV Co-Infection & Utility of Diagnostic Modalities in EPTB. Journal of clinical and diagnostic research : JCDR. 2013;7(8):1644-6. Epub 2013/10/03. doi: 10.7860/jcdr/2013/6363.3301. PubMed PMID: 24086863; PubMed Central PMCID: PMCPMC3782920.
47. Chakravorty SS, A. M.; Rowneki, M.; Parmar, H.; Cao, Y. et al. The New Xpert MTB/RIF Ultra: Improving Detection of Mycobacterium tuberculosis and Resistance to Rifampin in an Assay Suitable for Point-of-Care Testing. mBio. 2017;8(4). Epub 2017/08/31. doi: 10.1128/mBio.00812-17. PubMed PMID: 28851844; PubMed Central PMCID: PMCPMC5574709.
48. Shah M, Hanrahan C, Wang ZY, Dendukuri N. Lateral flow urine lipoarabinomannan assay for detecting active tuberculosis in HIV-positive adults. The Cochrane database of systematic reviews. 2016;(5):Cd011420. Epub 2016/05/11. doi: 10.1002/14651858.CD011420.pub2. PubMed PMID: 27163343; PubMed Central PMCID: PMCPMC4916932.
49. Gui X, Xiao H. Diagnosis of tuberculosis pleurisy with adenosine deaminase (ADA): a systematic review and meta-analysis. Int J Clin Exp Med. 2014;7(10):3126-35. Epub 2014/11/25. PubMed PMID: 25419343; PubMed Central PMCID: PMCPMC4238476.
50. Pandie S, Peter JG, Kerbelker ZS, Meldau R, Theron G, Govender U, et al. Diagnostic accuracy of quantitative PCR (Xpert MTB/RIF) for tuberculous pericarditis compared to adenosine deaminase and unstimulated interferon-gamma in a high burden setting: a prospective study. BMC Med. 2014;12:101. Epub 2014/06/20. doi: 10.1186/1741-7015-12-101. PubMed PMID: 24942470; PubMed Central PMCID: PMCPMC4073812.
51. Weber SF, Saravu K, Heller T, Kadavigere R, Vishwanath S. Point-of-Care Ultrasound for Extrapulmonary Tuberculosis in India: A Prospective Cohort Study in HIV-Positive and HIV-Negative Presumptive Tuberculosis Patients. Am J Trop Med Hyg. 2018;98(1):266-73. Epub 2017/11/17. doi: 10.4269/ajtmh.17-0486. PubMed PMID: 29141727; PubMed Central PMCID: PMCPMC5928723.
52. Di Tanna GL, Khaki AR, Theron G, McCarthy K, Cox H, Mupfumi L, et al. Effect of Xpert MTB/RIF on clinical outcomes in routine care settings: individual patient data meta-analysis. The Lancet Global health. 2019;7(2):e191-e9. Epub 2019/01/27. doi: 10.1016/s2214-109x(18)30458-3. PubMed PMID: 30683238; PubMed Central PMCID: PMCPMC6366854.

Supporting Information

Table S1 Definitions of Tuberculosis

Flowchart of patients included in KIULARCO

Click here to download Figure Fig 1..tif

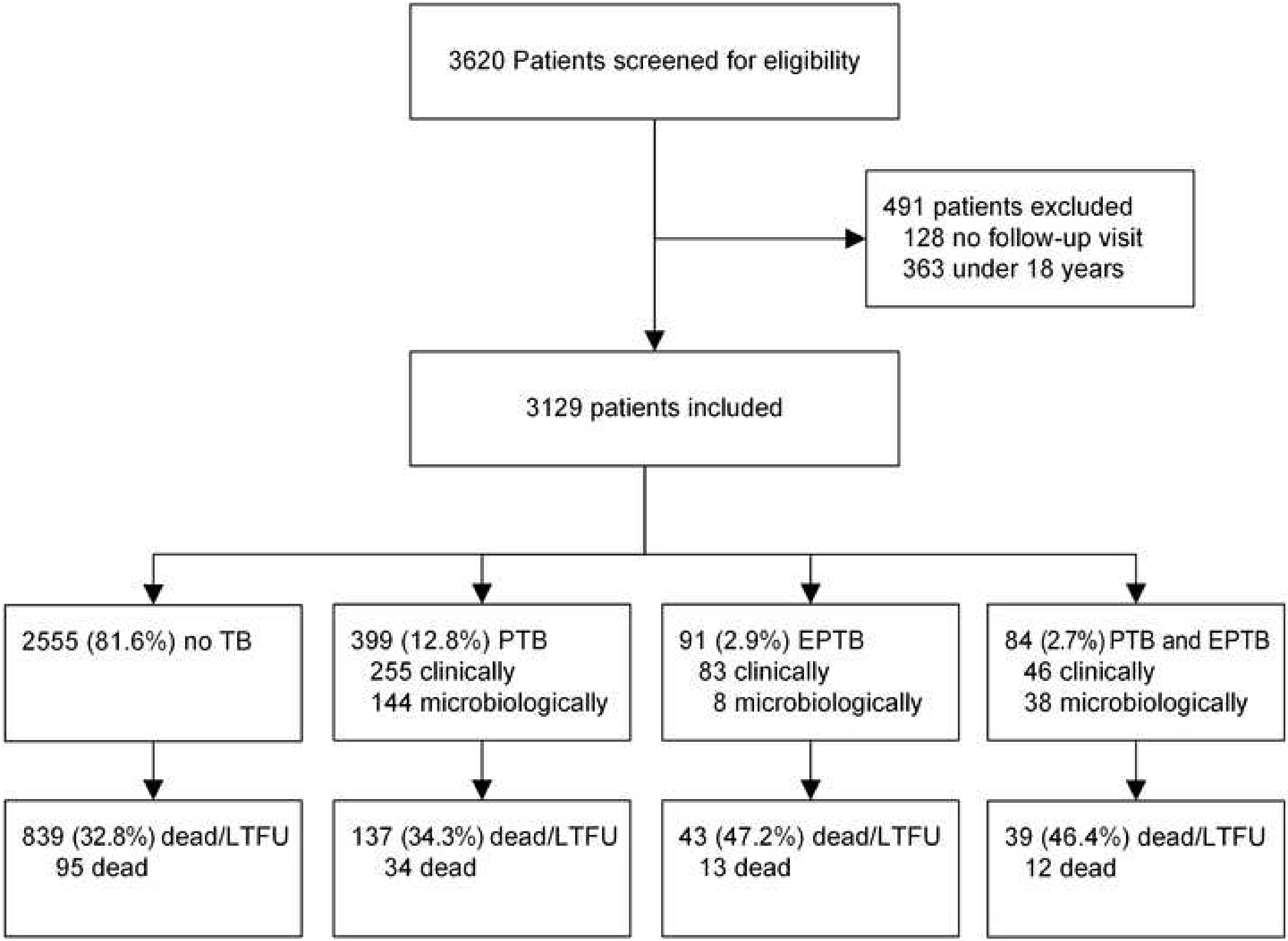

Intervall between diagnosis of EPTB and PTB and start of antiretroviral treatment

Click here to download Figure Fig 2..tif

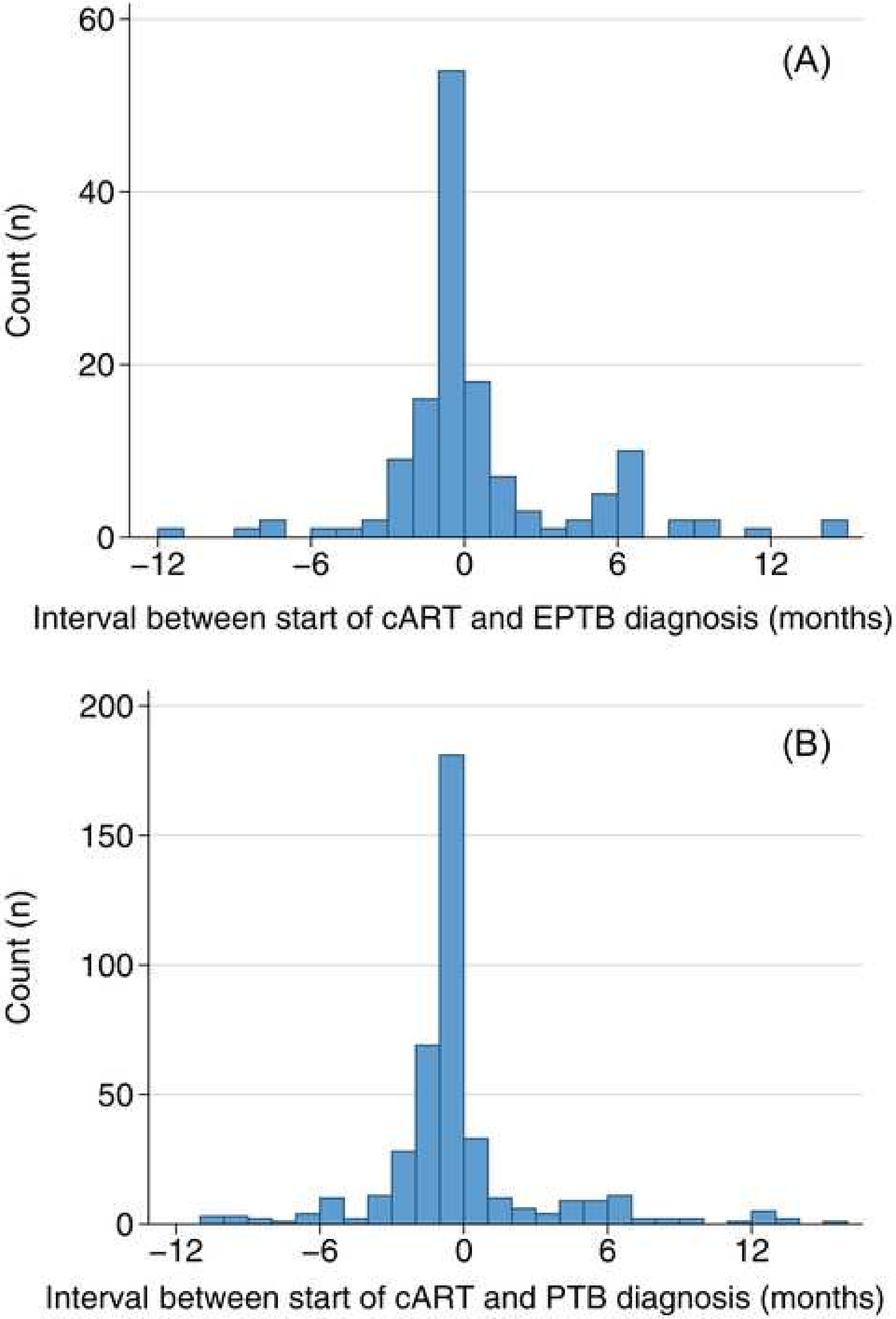

Kaplan-Meier curves for composite outcome of LTFU_death in Patients with tuberculosis

Click here to download Figure Fig 3..tif

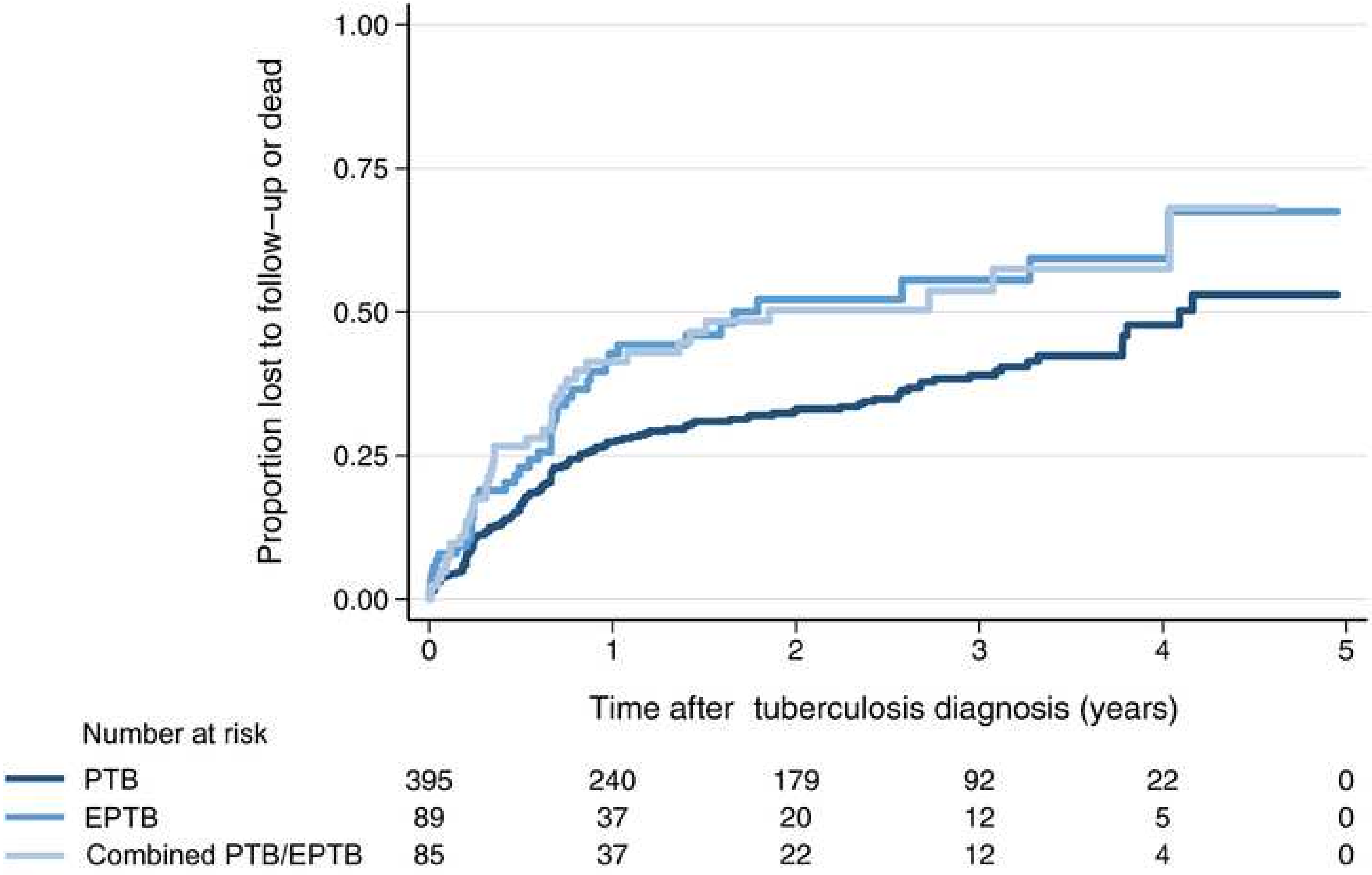

Table with definitions of tuberculosis

Click here to access/download
Supporting Information
S1 Table.docx

Dataset

Click here to access/download
Supporting Information
S2 Dataset.xlsx
