# Supplementary material for: Extrapulmonary tuberculosis in HIV-infected patients in rural Tanzania: The prospective Kilombero and Ulanga antiretroviral cohort
Source: PLoS One. 2020 Mar 4;15(3):e0229875. doi: 10.1371/journal.pone.0229875 (PMC7055864; doi:10.1371/journal.pone.0229875)
Supplement: Attachment — Submitted filename: Response to Reviewers.docx [file pone.0229875.s004.docx]

Marcel Yotebieng, M.D., MPH, Ph.D

Academic Editor

PLOS ONE

Ifakara, 11^th^ December 2019

**Resubmission of our manuscript entitled**
Extrapulmonary tuberculosis in HIV-infected patients in rural Tanzania. The prospective Kilombero and Ulanga antiretroviral cohort. PONE-D-19-26934

Dear Mr Yotebieng

On behalf of all co-authors I would like to thank you for the careful review process of our manuscript and for inviting us to resubmit a revised version to ***PLOS ONE.*** In the point-to-point reply attached below we addressed all comments of the reviewer and have modified the manuscript according to the suggestions made. Please find attached an updated version of the manuscript with highlighted changes. We feel, that these clarifications improved our manuscript and hope, it is acceptable for publication in its current form.

Yours sincerely

**Prof. Dr. med Maja Weisser**
*Clinical Research Coordinator of the Chronic Diseases Clinic of Ifakara; Tanzania*

*Ifakara Health Institute (IHI); Ifakara, Tanzania*

*Swiss Tropical & Public Health Institute (Swiss TPH); Basel; Switzerland*

*Infectious Diseases & Hospital Epidemiology, University Hospital Basel; Switzerland
e-mail: mweisser@ihi.or.tz
Phone: +255 62 102 95 69*

**Point to point reply**

**TITLE
Reviewer #1**
Instead of a full stop after Tanzania, a colon would be more appropriate as follows: Extrapulmonary tuberculosis in HIV-infected patients in rural Tanzania: The prospective Kilombero and Ulanga antiretroviral cohort.

**Reviewer #2**
1) I don’t understand why you have put “full stop” after Tanzania. Why not “:” ?

*ANSWER:* *We agree with this consideration and have changed the title accordingly.*

**ABSTRACT
Reviewer #1**
Reading through the paper, it appears that in addition to characterizing the epidemiology and clinical characteristics of these patients, the key outcomes of interest are death / loss to follow up rather than the broad term clinical outcomes which would also include outcomes such as cure. The authors should consider rewording the aim/objective to reflect the interest in the predictors of the outcome lost to follow up / death.

*ANSWER: We reworded to ‘risk factors of poor outcome’.*

**Reviewer #2**
1) Please you should explain all abbreviations at the first appearance in the text e.g: AFB, py, CI

*ANSWER: We thank for this suggestion and have corrected the abbreviations.*

2) You stated that: “The combined endpoint of death and LTFU was observed in 1058 (33.8%) patients, most frequently in the subgroup of EPTB (47.2%)”. You should give hazard ratio.

*ANSWER:* *We constructed a cox model exclusively for patients with EPTB, so we could investigate risk factors for the combined endpoint in these patients. Therefore, no hazard ratios for comparisons of different study groups (e.g. EPTB vs. PTB) can be indicated, as the cox model did never include patients of e.g. the PTB group. However, with the data given in Table 3, the frequencies of the combined endpoint in the different study groups can be compared. We added details on the test used for the calculation of p-values in the table legend, in addition to the preexisting mention in the methods section.*

3) “(HR 1.95, 95%CI 1.15-3.3; p = 0.013)”: This is not well written.
You can for example state after hazard ratio [HR (95% CI)] and then after each risk factor you just need to put the values.

*ANSWER: We agree with this proposal and adjusted the writing.*

**INTRODUCTION**

**Reviewer #1**
Page 4 Lines 80-81 My comment on the aim of the study highlighted under the ABSTRACT portion above also applies. The authors should consider rewording the aim/objective to reflect that they examined predictors of lost to follow up / death.

*ANSWER: We corrected as suggested (page 4, line 80).*

**Reviewer #2**
1) Line 55-56: “…14% incident cases” where?

*ANSWER:* *According to the Tuberculosis Report 2018 it applies to the incidence globally. We specified accordingly.*

2) Line 71: Heart failure is not the classical differential diagnosis of pleural TB. Heart failure gives typically a transudate pleural effusion whereas pleural TB gives exudative pleural effusion. You should remove heart failure as a differential diagnosis.

*ANSWER: We thank for this feedback. In remote rural settings, the laboratory mostly cannot provide a detailed analysis of fluid allowing differentiation between a transudate and an exudative character. Therefore, heart failure remains a possible differential diagnosis of pleural effusion. We have added the word ‘clinical’ to stress the constraint of diagnostic options.*

**METHODS

Reviewer #1**
Please indicate at the beginning of the Methods section that this is a prospective of study so that readers are aware of this methodology right from the start even before providing information on the study site and patient population. It would be helpful to readers to have a good understanding of the study setting since the authors highlight that it is rural Tanzania. So even though the authors provide references for details on the cohort, providing a little bit of information on the setting and the context of these patients would be very useful for readers especially later on when the results show such high rates of lost to follow up.

*ANSWER: We thank the reviewer for this comment and added more details in the highlighted manuscript on page 4 lines 85ff.*

Page 4 89-90 Please highlight what the guideline was for starting cART for HIV patients in the program.

*ANSWER:* *We added references for the guidelines used, which are the current “national*

*guidelines for the management of HIV and AIDS” (NACP), issued by the ministry of health of Tanzania.
During the study period the WHO guidelines recommendations and its derived NACP guidelines in start of antiretroviral drugs changed from HIV WHO stage III or IV or CD4 cell counts <350cells/mm3, to <500cells/mm3 to treatment of all HIV-positive individuals (page 4, line 93ff).*

Page 5 Lines 114-115 I found the following phrase “1 week before until 3 months after working diagnosis” challenging to understand. Is it to say that patients were designated as PTB even one week before they were assigned a diagnosis of TB? Please explain the phrase so that it is self-explanatory.

*ANSWER:* *We thank for this question. This sentence illustrates the difficulty that some patients started the treatment on a “probatory” base as the diagnosis of tuberculosis is often clinical only. Thus, the treatment might have started before the indication of an ICD-10 Code. We added working diagnosis of ‘suspected TB’ to make this clearer on page 5, line 120.*

Page 6 Lines 151-152 Please explain how the incidence density was calculated.

*ANSWER: The incidence density was calculated as (number of incident cases)/(person-time of subjects at risk in the unit of person-years). This contrasts with incidence rates (number of incident cases/numbers of subjects observed), which don’t incorporate a time dimension into the denominator. We added this in the definitions section on page 6, line 164.*

**Reviewer #2**
1) Line 101: Which sites (localizations) did you use for sonography screening? You should specify.

*ANSWER: We performed the sonography screening according to the Focused Assessment with Sonography for HIV and Tuberculosis protocol (FASH) and added the reference to the manuscript. The pathological findings screened for in the FASH-protocol were pleural or pericardial effusion, ascites, abdominal lymph nodes, hypoechogenic lesions in liver/spleen, Ileum wall thickening or ileum wall destruction (added on page 5, lines 109-111).*

2) The Methods section should begin by specify the group of patients included. You have put it at the end of the first paragraph.

*ANSWER:* *We thank for this correction, which was implemented in the manuscript.*

3) I don’t understand the rationale of “antituberculous drugs started” one week before the working diagnosis. This claim is confusing me.

*ANSWER: We thank the reviewer for this question. Please see our answer above to the same point raised by reviewer #1. Some patients started the treatment on clinical suspicion only, while microbiological prove follows later. We have added the explanation due to the possible premature start of TB-treatment before an indication of an ICD-10 diagnosis*

4) The section named definitions spanned data collection. You should rename or separate data collection and operational definitions sections.

*ANSWER:* *We reviewed the section entitled ‘definitions’ and believe the points mentioned should remain here as they are rather part of the analytical process than the prospective data collection.*

5) All the definitions used are very hard to understand and are a bit confusing. Can you specify the definitions used to create all the ICD10 codes?

*ANSWER:* *We agree it is a long paragraph, but we believe these definitions are key specifically for the field of the clinically suspected cases, which in daily life are done at the discretion of the treating physician. As we mentioned in the discussion this remains a limitation of the study. To simplify, we shortened the paragraph on definition of comorbidities on page 6, line 141 ff.*

6) The major issue in this chapter is the absence of histopathological features.

*ANSWER: We agree and have pointed this out in the methods page 5, line 116/117. Additionally, this is mentioned in the discussion as a limitation (page 14, line 336ff).*

7) In HIV environment we usually used 90 days for LTFU definition and not 60 days. What are the rationales of 60 days choice?

*ANSWER:* *When the definition for LTFU for use in this cohort was designed, we incorporated empirical evidence about the choice of the optimal cutoff to predict a true LTFU status. This study can be accessed at* [*https://www.ncbi.nlm.nih.gov/pubmed/20219765*](https://www.ncbi.nlm.nih.gov/pubmed/20219765) *.*

8) Which statistical test did you use to compare Kaplan-Meier curves (Log-rank test?)

*ANSWER: We added a p-value derived by log-rank testing to Figure 3, comparing the 3 groups of patients with a diagnosis tuberculosis, including a mention of the test in the methods section (page 6, line 163).*

*The p-values already indicated in the text comparing risk factors for the composite outcome in patients with EPTB are derived from the survival analysis using the Cox proportional hazard model. We think this was appropriately indicated by mentioning p-values in exclusively in parenthesis after the respective hazard ratios.*

**RESULTS**

*COMMENT: Please note the change in the inclusion timeline on page 7, line 178; we replaced September 2013 with January 2013, which was a mistake in the first edition.*

**Reviewer #1**
Page 7 Line 175 Please provide the range of the follow up period as well.

*ANSWER: Please find the range in the highlighted manuscript (3 days-5 years) on page 7, line 182.*

Page 7 Lines 179 – 188. The authors make statements such as “more advanced” and “no major differences”. It is not clear whether these inferences are being made from just eyeballing the figures Table 1. Please indicate whether these differences were statistically significant or not statistically significant.

*ANSWER: We thank for this comment and added the p-values of comparison tests (according to the methods section) to table 1.*

In Table 1, I noticed that registration was highest in 2015 and I am wondering whether there was some reason for this. The authors should please point out this finding and comment on it in the discussion.

*ANSWER: We thank you for the observation and discussed the finding with all investigators. However, we could not find a convincing conclusion to explain this peak. Therefore, we believe, this might reflect natural fluctuation or factors not explainable with the data on hand. We have already commented this in the discussion on page 13, line 289ff.*

Page 10 Line 209 For the incident TB cases, please indicate how many patients were diagnosed with TB 90 days after enrolment and the range of the period between the time of enrollment and the diagnosis of TB.

*ANSWER: Out of a total of 574 patients with TB (399 PTB, 84 PTB/EPTB and 91 EPTB), 334 PTB, 71 PTB/ETPB and 78 EPTB cases were diagnosed within the 3 months (baseline prevalence). Thus, 5 cases of PTB, 13 EPTB and 13 cases of EPTB/PTB were diagnosed after these first 3 months. We added this information on page 10, line 215-216.)*

*The range of days has been added in the manuscript on page 10, line 210 and 211.*

Please provide information on the prevalence of CNS manifestation in the EPTB patients.

*ANSWER: The prevalence rates of varying organ manifestations of EPTB were described in the initial manuscript under subheading “Clinical presentation and microbiological testing” on page 10, line 227. This includes CNS manifestations as well, which were present in 12% of all cases of EPTB.*

Please indicate what the reference groups are for all the variables in Table 4

*ANSWER: Reference groups were added as footnotes to the table as requested.*

**Reviewer #2**

1) What are the characteristics of 128 excluded patients in comparison of the characteristics of included ones?

*ANSWER: Those patients had high rates of missing values for most variables used in table 1, thus no clear description of these patients was possible. We added a comment in the manuscript on page 7, line 180.*

2) Did you verify that the patients classified as LTFU were not died?

*ANSWER: As LTFU commonly indicates a category of subjects, whose outcome is unknown, we cannot know if patients died. Indeed, a high death rate is described in studies actively tracking patients lost to follow-up (e.g. Egger et al* [*PLoS Med.*](https://www.ncbi.nlm.nih.gov/pubmed/21267057) *2011 Jan 18;8(1):e1000390): Correcting mortality for loss to follow-up: a nomogram applied to antiretroviral treatment programs in sub-Saharan Africa.) In our setting we do regular tracking, but still remain with a high percentage of patients LTFU.*

3) You have compared baseline characteristics in different groups without given p-value for comparison. The p-value is not specified in the Table 1.

*ANSWER: The p-values have been added to table 1.*

4) The first column in your Table 1 should be EPTB and you should compare the other forms to this one.

*ANSWER: We have changed the order as requested.*

5) Table 1 is not well formatted which horizontal lines inside the table.

*ANSWER: The table was indeed already designed to be legible without the necessity of horizontal lines. We removed the horizontal lines in the updated version.*

6) Line 199: You should begin by EPTB

*ANSWER: We thank for this correction and adjusted the title.*

7) Is it any difference between the median time from initiation of cART to the diagnosis of PTB and EPTB

*ANSWER: We added the p-value derived by a Mann–Whitney U test comparing the intervals to the results section, it was nonsignificant with a value of 0.78 (page 10, line 223).*

8) Line 222: I hope that the localization of EPTB was specified in all of your participants.

*ANSWER: In the 175 patients with an extrapulmonary manifestation the localization was always specified. The distribution of organ manifestations has already been described in the results section under “Clinical presentation and microbiological testing”.*

9) Lines 226 and 227: pulmonary and extrapulmonary manifestations refer to PTB and EPTB? Not clear.

*ANSWER: We used “manifestation” instead of “PTB and EPTB” to account for the fact that patients in both the EPTB and the combined PTB/EPTB study group show signs of extrapulmonary TB. The same is true for pulmonary manifestations which are present in the PTB and the combined PTB/EPTB group. The respective denominator in the numbers indicated in the same sentence is the appropriate sum of the two groups. We further clarified this in the methods (page 6, line 139).*

10) Table 2: please correct “Lymph node”

*ANSWER: We thank for the comment and corrected accordingly.*

11) Lines 240 and 241: please rephrase sentence. The second part is not understandable

*ANSWER: We agree with the comment and clarified the sentence (now page 11, line 248-249)*

12) Lines 250-251: EPTB and EPTB/PTB were compare to what (PTB or no TB)?

*ANSWER: Table 3 describes a crude comparison of outcomes in the four study groups. The indication of one p-value per row indicates that it describes a multiple group comparison across all 4 groups and not the result of an individual between-two-group comparison. We added a specification of this in the table legend (page 12, line 263).*

13) Figure 2 is difficult to understand. Negative month means what?

*ANSWER: The interval denotes the difference between date of diagnosis of TB and the initiation of cART in months. Positive numbers indicate, that the diagnosis of TB was after the initiation of cART, negative numbers indicate, that the diagnosis of TB was before the initiation of cART. We added a sentence in the figure legend (‘Out of the 574 patients with any diagnosis of TB, 508 (88.5%) started cART after a median of 23 (IQR 10-56) days.’)*

14) I did not see the verification of proportional hazard models assumptions**.**

*ANSWER: In the initial manuscript (“Data management and analysis”, page 6, line 165ff) we indicated, that Schoenfeld residuals were used to verify the proportional hazards assumption.*

*We are not sure, whether the reviewer was asking to actually see the results of the assumption testing done. In our opinion, it is good standard in medical literature to explicitly indicate that assumption testing was performed and to mention the respective method used. However, to our knowledge, the results of such testing are not commonly published with the article. If of interest for the reviewer, we show the results of the formal statistical testing here.*

| Variable | rho | chi2 | df | Prob>chi2 |
| --- | --- | --- | --- | --- |
| Sex | 0.08087 | 0.51 | 1 | 0.4755 |
| CD4+ cell count at baseline | 0.13093 | 1.33 | 1 | 0.2485 |
| Tuberculous meningitis | -0.18395 | 2.67 | 1 | 0.1022 |
| ART intake | -0.17803 | 3.14 | 1 | 0.0762 |
| Age > 45 years | 0.09997 | 0.83 | 1 | 0.362 |
|  |  |  |  |  |
| global test |  | 7.79 | 5 | 0.1683 |

**ACKNOWLEDGEMENTS**

*COMMENT: Page 16, line 356. We added the members of the KIULARCO study group in the acknowledgements. The corresponding author is Prof. Dr. Maja Weisser.*
