# Supplementary material for: Extrapulmonary tuberculosis in HIV-infected patients in rural Tanzania: The prospective Kilombero and Ulanga antiretroviral cohort
Source: PLoS One. 2020 Mar 4;15(3):e0229875. doi: 10.1371/journal.pone.0229875 (PMC7055864; doi:10.1371/journal.pone.0229875)
Supplement: Attachment — Submitted filename: Response to Reviewers.docx [file pone.0229875.s005.docx]

Marcel Yotebieng, M.D., MPH, Ph.D

Academic Editor

PLOS ONE

Ifakara, 4^th^ February 2020

**Resubmission of our manuscript entitled ‘Extrapulmonary tuberculosis in HIV-infected patients in rural Tanzania. The prospective Kilombero and Ulanga antiretroviral cohort’ (PONE-D-19-26934)**

Dear Mr Yotebieng

On behalf of all co-authors I thank you again for the new review of our manuscript and for inviting us to resubmit a revised version to ***PLOS ONE.*** In the point-to-point reply attached below we addressed the new comments of the reviewers and have modified the manuscript according to the suggestions made.

Please find attached an updated version of the manuscript with highlighted changes. We hope the manuscript is acceptable for publication in its current form.

Yours sincerely

**Prof. Dr. med Maja Weisser**
*Clinical Research Coordinator of the Chronic Diseases Clinic of Ifakara; Tanzania*

*Ifakara Health Institute (IHI); Ifakara, Tanzania*

*Swiss Tropical & Public Health Institute (Swiss TPH); Basel; Switzerland*

*Infectious Diseases & Hospital Epidemiology, University Hospital Basel; Switzerland
e-mail: mweisser@ihi.or.tz
Phone: +255 62 102 95 69*

**Point to point reply**

**GENERAL REMARKS**

*Please find a for table S1 in line 122, page 5.*

**Reviewer #1:**

The authors have done a good job addressing the points raised in the review. There are however a few outstanding minor points and some comments from the first review that the authors are kindly requested to address.

1. Please add ‘of suspected TB’ to the following phrase “1 week before until 3 months after working diagnosis” where it appears in the manuscript.

*We added accordingly (Line 135 and 138).*

2. The authors provide the following explanation to the reviewer comments “The incidence density was calculated as (number of incident cases)/(person-time of subjects at risk in the unit of person-years).” However, this definition/explanation should not only be for the benefit of the reviewer but should be included in the manuscript.

*We added this in the definitions section on page 6, line 164 ff.*

3. Page 7 Line 188: Please include the range in the following format “3 days-5 years” in the manuscript instead of 0.01-5.

*We adjusted the manuscript accordingly on page 7, line 190.*

4. Page 8 Line 198: The authors indicate “There were no major differences in marital status, education, smoking behavior and alcohol consumption”. However, the p-values for smoking, marital status are significant and there is no data for education in the Table. The authors should please address these discrepancies/gap.

*We corrected as suggested: ‘Patients with EPTB were more frequently married and less frequently smokers.’ (page 8, line 199 and table 1). We deleted the information on education.*

5. It looks like the following points from the initial review were not addressed by the authors so they should please address them:

DISCUSSION
The authors indicate a high mortality of 14.3% among their EPTB patients. How does this finding compare with what other studies have found?

*We added a section in the discussion, see page 14, line 302ff.*

Given the various variables that the authors report on in their study, there is opportunity for rich discussion on the study findings. The authors should include more discussion on the poor outcome of their patients LTFU in view of the clinical characteristics reported on such as WHO stage, comorbidities and CD4 count.

*The adjusted model did not show an association between indicators of HIV associated immunosuppression (WHO stage and CD4+ cell count) at baseline and the combined endpoint. This observation might be due to the inclusion of only patients developing EPTB into the model. By analyzing a selected group with a high probability of the endpoint as well as a presumed high level of functional immunodeficiency, WHO stage and CD4+ cell count might not further discriminate risk. Other parameters were not analyzed. We commented this on p 15, line 338 ff.*

In the conclusion, the authors highlight that lack of ART and age >45 years were risk factors for death or loss to follow-up. What recommendations do the authors offer for clinicians or program managers to address these findings?

*Lack of ART is an indicator of late presentation with insufficient time to start treatment in time, as ART initiation has to be postponed for 2-6 weeks in patients with EPTB not yet on ART. Therefore, the most important recommendation is early diagnosis of HIV and treatment start of ART. We adapted the conclusions on page 16, line 371 as follows: ‘The lack of ART as a factor for poor prognosis points at the importance of early HIV diagnosis and ART start’.*

*We abstained from giving recommendations for elder patients with an age >45 years. Firstly, we consider it difficult to give a recommendation based on an observational study, secondly there might be other confounders such as other age-related diseases increasing the mortality. Please see page 16, line 367*

**Reviewer #2:**

ABSTRACT
You stated that: “The combined endpoint of death and LTFU was observed in 1058 (33.8%) patients, most frequently in the subgroup of EPTB (47.2%)”. You should give hazard ratio.
ANSWER: We constructed a cox model exclusively for patients with EPTB, so we could investigate risk factors for the combined endpoint in these patients. Therefore, no hazard ratios for comparisons of different study groups (e.g. EPTB vs. PTB) can be indicated, as the cox model did never include patients of e.g. the PTB group. However, with the data given in Table 3, the frequencies of the combined endpoint in the different study groups can be compared. We added details on the test used for the calculation of p-values in the table legend, in addition to the preexisting mention in the methods section.
GENERAL COMMENTS
I don’t agree. When you used cox-model you should compare groups of patients in the cohort. The comparison of the group with outcome to the one without outcome would give the hazard of the outcome e.g: HR should be specified.
As I stated, the major problem of the paper is the absence of methods to roll out other differential diagnosis (no cytological or histological examinations).

1) *As your comment was pointing to results from table 3 where all study groups (i.e. including those with no TB) are summarized, we assumed you were also asking for HRs from a survival model of all patients in the study. As we explained in the previous comment and in the manuscript, we did not build a cox model including e.g. patients with no TB. This selection is based on the study question of finding risk factors in patients with EPTB. A model including also patients without any TB episodes would be much more complex (e.g. TB diagnosis would have to be modelled as a time-dependent covariate) and less interpretable without bringing any advantage for the answer of the study question.*

*However, we understand you comment as an indication for the need for a relative measure to compare the rate of the primary outcome in the patient groups with any TB as in Figure 3. To address this, we build a model including the PTB, EPTB and the EPTB/PTB groups and indicated HRs comparing those groups in the results section (p, line). This information replaces the results of the log-rank test in Figure 3 (added in the last round of review) in a more detailed way, so we removed it again.*

*In case this is not the information you were expecting, please specify in more detail the type of analysis to be conducted.*

*Abstract page 2, line 43. Results page 12, line 265.*

|  | **HR** | **95% CI** | **p** |
| --- | --- | --- | --- |
| PTB | Reference |  |  |
| EPTB | 1.63 | 1.14-2.31 | 0.006 |
| EPTB and PTB | 1.65 | 1.15-2.36 | 0.006 |

2) *We agree, that the absence of methods to roll out other differential diagnosis is a limitation of the study. We underline this in the discussion on page 16, line 349ff.*
